# Uveoscleral Outflow Routes after MicroPulse Laser Therapy for Refractory Glaucoma: An Optical Coherence Tomography Study of the Sclera

**DOI:** 10.3390/ijms25115913

**Published:** 2024-05-29

**Authors:** Luca Agnifili, Andrea Palamini, Lorenza Brescia, Annamaria Porreca, Francesco Oddone, Lucia Tanga, Maria Ludovica Ruggeri, Alberto Quarta, Rodolfo Mastropasqua, Marta Di Nicola, Leonardo Mastropasqua

**Affiliations:** 1 Ophthalmology Clinic, Department of Medicine and Ageing Science, “G. d’Annunzio” University Chieti-Pescara, 66100 Chieti, Italybrescia.lorenza@gmail.com (L.B.);; 2 Laboratory of Biostatistics, Department of Medical, Oral and Biotechnological Sciences, “G. d’Annunzio” University Chieti-Pescara, 66100 Chieti, Italy; annamaria.porreca@unich.it (A.P.);; 3 IRCCS Fondazione Bietti, Via Livenza, 3, 00198 Rome, Italy; 4 Department of Neuroscience, Imaging and Clinical Science, “G. d’Annunzio” University Chieti-Pescara, 66100 Chieti, Italy

**Keywords:** refractory glaucoma, uveoscleral aqueous humor outflow, MicroPulse transscleral laser treatment, MP-TLT, anterior segment-OCT, intraocular pressure

## Abstract

To analyze in vivo scleral changes induced by MicroPulse transscleral laser therapy (MP-TLT) in refractory glaucoma using anterior segment–optical coherence tomography (AS-OCT). Forty-two candidate patients for MP-TLT were consecutively enrolled and underwent AS-OCT at baseline and after six months. MP-TLT success was defined as an intraocular pressure (IOP) reduction by one-third. The main outcome measures were the mean superior (S-), inferior (I-), and total (T-) intra-scleral hypo-reflective space area (MISHA: mm^2^) and scleral reflectivity (S-SR, I-SR, T-SR; arbitrary scale) as in vivo biomarkers of uveoscleral aqueous humor (AH) outflow. The IOP was the secondary outcome. The relations between the baseline-to-six months differences (D) of DS-MISHA, DI-MISHA, and DT-MISHA and DS-SR, DI-SR, DT-SR, and DIOP, were investigated. At 6 months, the median IOP reduction was 21% in the failures and 38% in the successes. The baseline S-MISHA, I-MISHA, and T-MISHA did not differ between the groups, while S-SR and T-SR were higher in the successes (*p* < 0.05). At six months, successful and failed MP-TLTs showed a 50% increase in S-MISHA (*p* < 0.001; *p* = 0.037), whereas I-SR and T-SR reduced only in the successes (*p* = 0.002; *p* = 0.001). When comparing DS-MISHA, DI-MISHA, and DT-MISHA and DS-SR, DI-SR, and DT-SR, there were no significant differences between the groups. In the successful procedures, DIOP was positively correlated with DT-MISHA and DI-MISHA (ρ = 0.438 and ρ = 0.490; *p* < 0.05). MP-TLT produced potentially advantageous modifications of the sclera in refractory glaucoma. Given the partial correlation between these modifications and post-treatment IOP reduction, our study confirmed that the activation of the uveoscleral AH outflow route could significantly contribute to the IOP lowering after MP-TLT.

## 1. Introduction

Glaucoma is a chronic and multifactorial optic neuropathy, with intraocular pressure (IOP) reduction being the only proven strategy to slow the rate of damage progression [1,2]. When medical or laser treatments fail, surgical strategies are required to reach the target IOP [3]. Unfortunately, many patients can be refractory to standard surgical procedures, producing repeated failures [4,5]. These cases could benefit from surgical approaches that reduce the production of aqueous humor (AH) and/or promote unconventional drainage outflow pathways but avoid AH sub-conjunctival shunting. Cyclo-ablative procedures are suitable options in these cases [6]. However, because of potential serious sight-threatening complications, these procedures are recommended with caution and are generally used in eyes with poor visual acuity (VA) [7]. In the last few years, less-invasive treatments have been developed that have less of an effect on untargeted tissues [8]. MicroPulse transscleral diode laser cyclophotocoagulation (MP-TSCP) is the most recently introduced cyclo-ablative procedure for glaucoma. Since pulses of an active diode laser are interspersed with rest periods, the OFF cycles avoid coagulation necrosis of the ciliary body (CB), permit heat dissipation, and reduce the occurrence of undesired effects [9]. Hence, the definition of this procedure as MicroPulse transscleral laser treatment (MP-TLT) rather than MP-TSCP appears more appropriate and is currently adopted [10].

The IOP reduction after MP-TLT probably results from multiple mechanisms of action involving both the inflow and outflow AH pathways [11,12,13,14]. Tsujisawa et al. observed that MP-TLT reduces the AH inflow by damaging non-pigmented epithelial cells of the pars plicata and basal infoldings destruction [14]. Johnstone observed that MP-TLT shortens the CB and enlarges the trabecular spaces, thus favoring the conventional AH outflow [12]. Barac and coworkers documented a choroidal thickness increase three months after successful MP-TLT, thus indicating an increase in the AH uveoscleral outflow (AHUO) [15].

To date, it has not been investigated whether MP-TLT induces changes in the sclera, which is the terminal part of the AHUO pathway, nor if these changes contribute to overall IOP lowering. Previous anterior segment–optical coherence studies (AS-OCTs) conducted on patients undergoing ultrasound ciliary plasty (UCP) found that the formation of post-operative hypo-reflective spaces within the scleral stroma, along with the reduction in the scleral reflectivity, can be considered as surrogate in vivo biomarkers of an increased AHUO [16,17]. To explore the effects of MP-TLT on the AHUO routes differently, the present in vivo study analyzed the scleral features before and after MP-TLT using AS-OCT. It investigated whether scleral changes correlated with the IOP reduction in patients with refractory glaucoma.

## 2. Results

Overall, 81% of patients in this study underwent one previous glaucoma filtration surgery, 11% underwent two filtration surgeries, and 8% underwent a tube implantation (Ahmed valve implant) after a failed filtration procedure. No significant differences were found for age (Fs = 69.0 [57.0;77.0], Ss = 72.0 [66.5;78.0]; *p* = 0.312), gender (F/M: 7/12 and 14/9; *p* = 0.215), and previous glaucoma procedures (FS: 42.1% vs. SS: 52.2%, *p* = 0.734) between the Ss and Fs. The baseline VF damage did not differ between the groups, with MD values of −14.81 ± 1.21 dB and −15.23 ± 1.15 dB in the Ss and Fs, respectively (*p* > 0.05). The MD did not significantly change at the last follow-up, neither in the Ss nor Fs (−15.07 ± 1.24 dB and −15.39 ± 1.16 dB, respectively).

The MP-TLT was successfully completed in all patients, and none underwent re-treatment during the follow-up. Intra-operative complications were not reported in any of the cases, while post-operative complications were mild and transient without significant differences between the Ss and Fs (Table 1). None of the patients required adjunctive glaucoma surgery during the study period.

**Figure 1:**
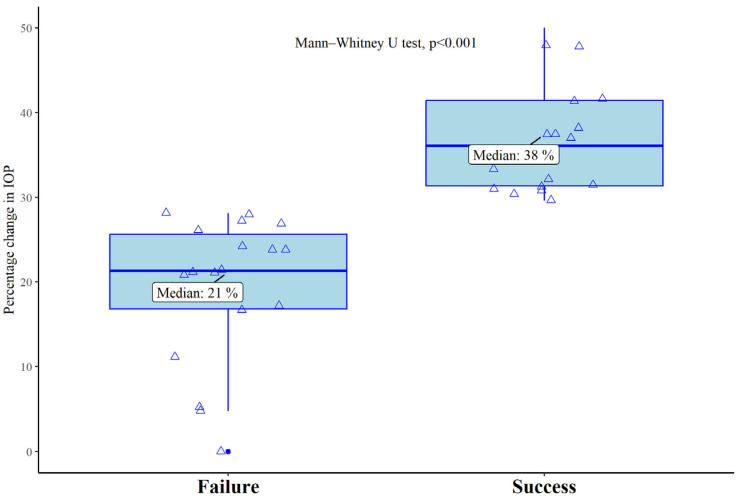
Boxplot for relative percentage change in IOP between baseline and six months in Fs and Ss. IOP: intraocular pressure (mmHg).

The overall success rate of the MP-TLT was 54.7% (23/42), with 60.9% in females (14/21) and 39.1% in males (9/21). In the 6 months, the median IOP reduction was 21% in the Fs and 38% in the Ss (Figure 1). There were no significant differences in the mean number of pre-operative and six months IOP-lowering medications in the Fs (2.89 ± 1.08 and 2.79 ± 1.12), whereas they significantly reduced in the Ss (2.85, 0.98 SD and 2.31, 1.02 SD; *p* < 0.05).

**Figure 2:**
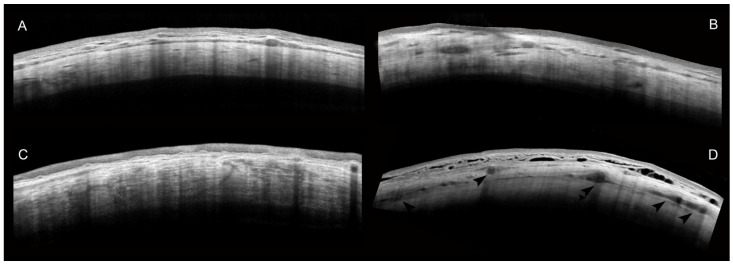
Mosaic of AS-OCT images representing the scleral modifications after the MP-TLT in the successful and failed procedures. In the left side of the image, (**A**,**C**) represent the pre-treatment scleral stroma features in the Fs and Ss, respectively. The right side of the image shows the scleral changes six months after the MP-TLT. In the Fs (**B**), the SR did not change, whereas the MISHA increased; in the Ss (**D**), there was a higher increase in the MISHA in the middle stroma (arrowheads) and a general reduction in the SR.

**Figure 3:**
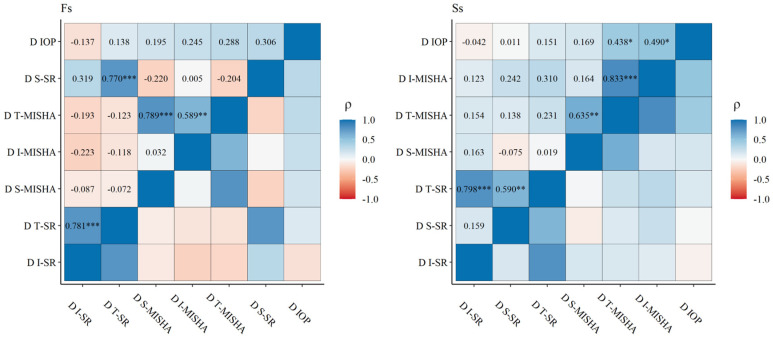
The correlation matrix using the listwise deletion method for the Fs and Ss groups. The matrix reports Spearman’s correlation coefficients (ρ) and significance level codes: * *p* < 0.05, ** *p* < 0.01, *** *p* < 0.001. IOP (mmHg); I-, S-, and T-MISHAs: inferior, superior, and total mean intra-scleral hypo-reflective areas (mm^2^); I-SR, S-SR, and T-SR: inferior, superior, and total scleral reflectivities (arbitrary grading scale). DIOP, D I-MISHA, S-MISHA, T-MISHA, D I-SR, S-SR, and T-SR are baseline-to-six months differences (D).

The baseline IOP and AS-OCT parameters are reported in Table 2: no significant differences were found for the IOP; S-, I-, T-MISHA; and I-SR between the Ss and Fs, whereas the S- and T-SR were significantly higher in the Ss (*p* < 0.05). In the Fs, the SR had no significant modifications six months after the MicroPulse treatment, while S-MISHA increased (*p* = 0.037). The Ss had a 50% increase in S-MISHA (*p* < 0.001). In contrast, the I-SR and T-SR reduced by almost 10% (*p* = 0.002 and *p* = 0.001, respectively) (Figure 2). When comparing the DS-MISHA, DI-MISHA, and DT-MISHA and the DS-SR, DI-SR, and DT-SR, there were no significant differences between the Fs and Ss (Table 3). Figure 3 shows the correlations between the IOP change (DIOP) and AS-OCT parameter changes: in successful procedures, the DIOP was positively correlated with the DT-MISHA and DI-MISHA (ρ = 0.438 and ρ = 0.490, respectively; *p* < 0.05).

## 3. Discussion

Given that the laser beam impacts the non-secreting part of the CB, the AH transport dysfunction probably does not represent the unique (or the main) mechanism of action exploited by MP-TLT to lower the IOP [10]. Histological studies on enucleated cadaver eyes, which compared the tissue effects of MP-TLT versus continuous-wave (CW)-TSCPC, showed that the destruction of the secretive CB epithelium was induced by CW-TSCPC but not by MP-TLT [13].

On the other hand, as mentioned above, Barac and coworkers observed that successful MP-TLT produced a 7.3% increase in the choroidal thickness sustained until the third month [15]. Moreover, a post-treatment increase in spaces between ciliary muscle bundles was also reported [11,12]. All these findings indicate that MP-TLT could have a minimal impact on the AH inflow, while the increase in outflow could be a major mechanism for lowering the IOP.

The results of our study seem to confirm, at least partly, the involvement of the unconventional AH routes in the overall IOP lowering induced by the MP-TLT. In more detail, we observed that six months after the successful MP-TLT, the sclera presented as more loosely arranged, as indicated by the 50% increase in hypo-reflective spaces and the small reduction in tissue reflectivity. The increased presence of optically clear spaces and the attenuation of tissue reflectivity can be intended as AS-OCT markers of scleral fibers delamination and collagen loss and, therefore, the expression of reduced tissue resistivity to fluid movement along the AHUO route.

Such favorable scleral remodeling is in line with what happens after a UCP, which is a different ab-externo cyclo-ablative procedure that, besides the ciliary epithelium necrosis, increases the AHUO. Previous evidence showed that when a UCP is successful, there is a two-to-threefold increase in the MISHA, which significantly correlates with the IOP reduction [16]. Though the nature of the energy delivered by MP-TLT and UCP differs, it is hypothesized that in both treatments, the probe creates a halo that favorably impacts tissues composing the AHUO route, such as the sclera and supra-choroid. This hypothesis was taken into consideration by Tan et al. in a study that investigated the effects of MP-TLT on rabbit conjunctiva [18]. Since the procedure induced inflammation and fibrosis of the bulbar conjunctiva, they proposed that the laser energy extends its effects on neighbouring tissues, promoting changes to the conjunctiva and deeper into the sclera.

However, some recent experimental findings do not seem to be particularly in line with our results. MP-TLT was reported to induce an upregulation in collagen and fibronectin and stimulate the formation of short and tortuous collagen fibers within the rabbit sclera [19]. Though this study confirmed that the MP-TLT stimulated the AHUO by up-regulating the expression of matrix-metalloproteinases 1–3 within the CB, the induction of fibrotic processes could lead to an unfavorable remodeling of the sclera, which may hamper the intra-tissue fluid movement. Nevertheless, we must consider that the rabbit sclera is approximately half as thick as the human sclera, that the study was performed on healthy eyes, and that it had a very short follow-up (one week) [18]. The latter is crucial since long-term tissue remodelling, such as that explored in our study, could significantly differ from short-term/acute tissue changes. As a potential further support to our findings, Tsujisawa et al. did not observe signs of scleral thinning one week after MP-TLT, which seems to indirectly rule out phenomena of tissue dehydration (and increased fluid resistivity) in the early phases [14].

Surprisingly, while the reduction in the SR was an exclusive feature of the Ss, a significant increase in the S-MISHA was also found in the Fs (although with low statistical significance). A possible explanation is that scleral delamination could happen in all eyes undergoing MP-TLT; however, it could only lead to a significant IOP reduction when coupled with additional stromal changes, such as a collagen amount reduction (indirectly expressed by the SR reduction). As stated, this feature could not appear in the early phases after treatment, where acute changes predominated (and the increase in the AHUO depended on the effects of inflammatory mediators). Still, it could become evident only in the long term [19].

However, our results should be interpreted with caution. Though in successful cases, the S-MISHA and S- and T-SRs significantly increased, we did not find differences between the groups when comparing the baseline-to-six-month data. This means that we observed modifications of the sclera that happened only when the procedure lowered the IOP. Still, we cannot state that these modifications are biomarkers that discriminate successes from failures.

Nevertheless, the potential advantageous effects of scleral changes on the IOP control were supported by the significant correlations between the DIOP, DT-MISHA, and I-MISHA only in successful cases. This means that when there was a greater amount of IOP reduction, there was a greater increase in the area of the hypo-reflective space within the stromal sclera. Given that the formation of hypo-reflective spaces can be intended as imaging biomarkers of increased intra-scleral fluid movement, one may speculate that MP-TLT requires the stimulation of unconventional outflow routes to reduce the IOP effectively.

Moreover, we found evident differences in the MP-TLT’s success between the two sexes, with a higher success rate in females than in males. This could probably depend on some gender-related differences in the scleral architecture. Tehrani et al., after reviewing the literature, found that there are genetics-related factors, anatomical variations, epidemiological features, and endocrinological discrepancies that may account for a different response to treatments between males and females [20]. Consistently, Kramer et al. reported physical differences in the sclera and episclera between males and females regarding vascularization, redness, and thickness [21].

Finally, after checking the literature for long-term studies on MP-TLT effects, we found that our results generally align with current evidence. Moctar et al. analyzed 39 eyes of 31 patients with a diagnosis of refractory glaucoma, reporting a 60.5% overall success rate 11 months after the procedure [22]. Similarly, Zemba et al., after comparing MicroPulse and continuous-wave transscleral cyclophotocoagulation in refractory neovascular glaucoma in a 12-month follow-up study, reported a similar efficacy of the two techniques in terms of IOP lowering, with a lower safety profile of the continuous-wave procedure [23]. Finally, Garcia et al., in a six-month study, confirmed the IOP-lowering effects and good safety profile of MP-TLT for refractory glaucoma [24].

However, some limitations must be pointed out. First, a potential limitation was the before–after design of this study. As is known, this design could introduce biases since the variations observed could depend on concomitant events that occurred around the same time as the intervention. However, one may suppose that in this specific case, it is highly improbable that scleral changes, which are an expression of deep tissue remodelling, would have occurred spontaneously or because of concomitant unknown events. Nevertheless, a comparator arm would have permitted evaluation of whether scleral effects induced by the MP-TLT differed from those produced by other myeloablative procedures or whether the sclera of unoperated eyes underwent the same modifications observed in a previously manipulated sclera. Second, we did not directly measure the AHUO, but we evaluated surrogate morphological aspects, which probably indicate a tissue remodeling that facilitated the fluid resorption. Thus, we cannot be sure that scleral changes were clear signs of a uveoscleral outflow increase. However, given that such scleral features were previously proposed as AHUO biomarkers in a similar CB procedure (the UCP) and also in patients with rhegmatogenous retinal detachment [25], in which AHUO clearly increased, we think that our hypothesis is reasonable. Third, since AS-OCT cannot visualize structures located beyond the iris pigmented epithelium, we could not assess the effects of the procedure on the CB. Therefore, we cannot state the real contribution of the AHUO increase in the final IOP lowering. Fourth, since our sample was composed of patients with an advanced stage of disease, we cannot extend our findings to mild-to-moderate glaucoma. Fifth, given that we exclusively considered refractory glaucoma, our findings cannot be generalized to patients who did not undergo previous surgeries. One may, in fact, presume, as anticipated above, that unoperated eyes have different anatomical features of the sclera, which, then, may react differently to the MicroPulse laser.

To summarize, the present in vivo study, which was the only mid-to-long-term pathophysiological study on MP-TLT in humans, confirmed the favorable effects of this treatment on the AHUO pathways. Considering the beneficial effects of MP-TLT on outflow pathways, the procedure can be considered as a valid, effective, and safe alternative to other destructive cycloablative procedures in refractory glaucoma. When considering the potential MP-TLT’s impact on currently utilized treatment protocols and on future strategies of glaucoma management, one may consider the procedure to be a suitable first-line approach in selected cases (e.g., the impossibility to undergo filtration procedures due to higher risk for potential sight-threatening complications, particular eye characteristics, or a lack of compliance with follow-up examinations).

To conclude, these results, which suggest the presence of a stimulative (on outflow routes) rather than an exclusively demolitive (on inflow routes) effect of MP-TLT, further support the potential indication of this therapeutic approach, not only for refractory glaucoma but also for patients who did not undergo previous filtration surgery [26,27]. However, further imaging studies are required to explore whether MP-TLT produces the same changes in naïve-to-surgery sclera, and what are the differences with respect to other cycloablative procedures, if any.

## 4. Materials and Methods

### 4.1. Patients’ Enrolment

For this observational, prospective study, patients were asked to sign an informed consent form that reported the nature and possible consequences of the study before enrolment. The study was approved by our institutional review board (Department of Medicine and Ageing Science of the University ‘G. d’Annunzio’ of Chieti-Pescara, Chieti, Italy), and it complied with the principles of the Declaration of Helsinki.

Forty-two consecutive Caucasian patients scheduled to undergo MP-TLT for uncontrolled refractory glaucoma were enrolled at the Glaucoma Center of the University ‘G. d’Annunzio’ of Chieti-Pescara. At baseline, the patients underwent best-corrected VA determination, Goldmann applanation tonometry, un-dilated fundus examination, and a visual field test (24-2 test, full threshold, HFA III; Carl Zeiss Meditec, Inc., Dublin, CA, USA) to stage the glaucomatous damage (Hodapp-Parrish-Anderson criteria) [28].

Inclusion criteria were age ≥ 18 years, uncontrolled IOP (>21 mmHg, 9 AM) or progression of glaucomatous perimetric damage, refractory glaucoma with at least one failed previous incisional surgery, and maximally tolerated medical therapy. Maximal medical therapy is required using all tolerable IOP-lowering eye drops and oral acetazolamide when needed. Visual field (VF) damage progression was evaluated through trend analysis using the HFA Guided Progression Analysis software (software version 1.5.3.714 accessed on July 2023). The progression was considered clinically significant when the VF index slope was worse than 1% per year with a *p*-value ≤ 0.05. The exclusion criteria were the following: history of previous ciliary body ablative procedures or retinal detachment, surgical or laser procedures to reduce the IOP performed in the last two months, previous ocular trauma with ciliary body or scleral involvement, presence of regions of scleral thinning, end-stage glaucoma, and pregnancy. If both eyes were eligible for the study, the eye with the higher IOP was included; if the IOP was similar in both eyes, the eye with the more advanced stage of disease was enrolled. The MP-TLT was considered successful when a reduction of at least 30% in the preoperative IOP was achieved at the 6-month follow-up with the maintenance of IOP-lowering medications during the entire follow-up.

### 4.2. Surgical Procedure

The MP-TLT procedure and the technical characteristics of the device were previously described [29]. To perform the treatment, which was delivered under local anesthesia, we utilized the Cyclo Glaucoma Laser System (IRIDEX Corporation, Mountain View, CA, USA) with the MicroPulse P3 probe (810 nm infrared diode) (version 1). The laser power was set between 2000 and 2500 mW and delivered with a duty cycle of 31.3%, equivalent to 0.5 ms of “on time” and 1.1 ms of “off time”. The MP-TLT was performed throughout 360 degrees, with the probe applied perpendicularly to the scleral plane and, to target the pars plana, 3 mm posteriorly to the limbus. If there were flat filtration blebs, this region was included in the treatment; encapsulated blebs were avoided. Nasal and temporal sectors (3 and 9 o’clock) were not treated to avoid inadvertent damage to ciliary arteries. The probe was held with a steady pressure over the conjunctiva during the treatment. It was moved continuously around the peri-limbal circumference in a painting manner, with slow back-and-forth sliding. Sweeps were performed four times in the superior hemisphere and four times in the inferior hemisphere; each sweep in one direction took approximately 20 s (thus, 80 s for each hemisphere), for an approximate total duration of treatment of 160 s.

As per protocol, the post-operative treatment included a topical betamethasone 2 mg/mL and chloramphenicol 5 mg/mL fixed combination (Betabioptal; Théa Laboratoires, Clermont-Ferrand, France) given for one week, three times a day. During follow-up, pre-operative hypotensive medications were maintained, but the therapy regimen could be modified according to the IOP values. Early post-operative visits included safety checks on days one and seven, with BCVA determination, tonometry, slit lamp biomicroscopy, and a fundus examination. For each case, adjunctive surgical treatments were allowed if the IOP did not reach the desired target value in the first month and the patient abandoned the study.

### 4.3. Examinations

#### AS-OCT of the Sclera

The RTVue XR Avanti (Optovue, Inc., Fremont, CA, USA) was the AS-OCT platform used for the present study. AS-OCT was performed the day before the MP-TLT and six months later to analyze the mean intra-scleral hypo-reflective spaces area (MIHSA, mm^2^) and the scleral reflectivity (SR).

Ten 6 mm long vertical cross-sectional scans were performed in six scleral sectors. Three sectors were analyzed in the inferior sclera (4, 6, and 8 o’clock) and three superiorly. Three unmanipulated regions were evaluated in the superior sclera to avoid the analysis of scleral sectors modified from previous filtration surgeries or tube implantation. The corresponding scleral regions were not investigated since MP-TLT does not treat nasal and temporal sectors. The scan selection was set to automatic mode so that the video brightness and contrast were standard. Three high-quality scans (from 30 randomly selected images) were chosen from each of the six regions by one operator (A.P.), who also carried out all examinations. After evaluating images, a second operator (L.B.) averaged the results to analyze the superior and inferior sectors and the entire sclera.

**Figure 4:**
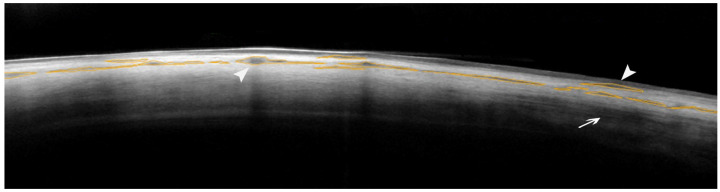
AS-OCT image showing the identification and manual demarcation of the hypo-reflective areas within the scleral stroma to calculate the MISHA. Areas of significant scleral hypo-reflectivity are outlined in ocher. White arrowheads indicate examples of areas of scleral hypo-reflectivity, whereas the arrow indicates an area of undefined hypo-reflectivity, which was not considered when calculating the MISHA. In this representative case (super-temporal sector of a right eye), the sum of the areas of scleral hypo-reflectivity produced a MISHA value of 0.0117 mm^2^ (Image J).

The MISHA and SR were calculated with Image J, which is an open-source software for processing and analyzing scientific images (Image J software, version 1.52°, http://rsb.info.nih.gov/nih-image, accessed on 1 September 2023). As previously defined, intra-scleral hypo-reflective spaces are intra-stromal features in which the sclera presents well-defined areas with less intense reflectivity than the surrounding regions [16,25]. These hypo-reflective spaces should be intended as areas where the scleral stroma is loosely arranged and less compact and, therefore, with probably lower hydraulic resistivity. Since variations in the scleral reflectivity (which depend on the number of collagen fibers and their disposition) can normally be found, only hypo-reflective areas with well-defined limits and with a mean grey value lower than 50% of the mean grey value of the surrounding sclera were considered as MP-TLT-related features (Figure 4).

**Figure 5:**
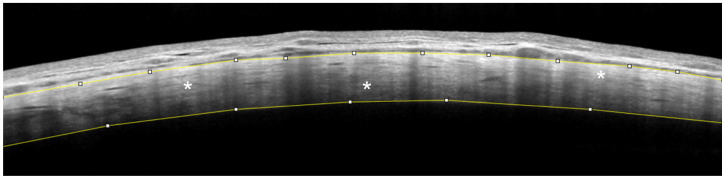
AS-OCT image showing the determination of the scleral stroma reflectivity. In this representative case (infero-nasal sector of a right eye), the upper and lower yellow lines, manually outlined, demarcate the margins of the scleral stroma. White asterisks indicate the three stromal regions (in the middle–outer scleral layers), which were selected to calculate the mean reflectivity of the sclera (mean value of 196.034, Image J).

The SR indicates the number of collagen fibers within the scleral stroma. Thus, when the sclera shows a hypo-reflective aspect in each region, this probably indicates the presence of loosely arranged and less dense tissue with a higher hydraulic conductivity. To determine the SR, we selected three homogeneous and hypo-reflective-space-free areas (100 × 100 µm) of the middle–outer sclera in the three high-quality AS-OCT scans (Figure 5). The Image J software calculated the average grey value of the selected regions. The result equaled the total sum of all pixels’ grey values divided by the total number of pixels. To ensure a re-analyzation of the sixth month, the same sectors in methylene blue were used at baseline to mark the conjunctiva overlying the sclera in the selected regions. Afterward, a set of reference photographs was acquired to guide the operator in the follow-up analysis.

For both the MISHA and SR, we calculated the averages of the superior and inferior sectors, and the total average (S-MISHA, I-MISHA, and T-MISHA and S-SR, I-SR, and T-SR). The MISHA and SR were the primary outcomes; the IOP was the secondary outcome. The relations between the baseline-to-six months differences (Ds) of S-MISHA, I-MISHA, and T-MISHA; S-SR, I-SR, and T-SR (DS-MISHA, DI-MISHA, and DT-MISHA and DS-SR, DI-SR, and DT-SR); and DIOP were investigated.

### 4.4. Statistical Analysis

The descriptive statistics are expressed as the absolute frequency and percentage (%) for categorical variables and as median [q1 = first quartile, q3 = third quartile] for continuous variables. The normality assumption was tested using the Jarque–Bera test. The correlation matrix using Spearman’s ρ correlation coefficient with listwise deletion was computed to assess the relationship between the continuous parameters in the successes (Ss) and failures (Fs). The Wilcoxon rank sum test was used to reveal the differences over time (from baseline to 6 months) into groups. The Mann–Whitney U test was applied to investigate whether significant differences existed between the groups at baseline, at 6 months, and between the percentages of IOP reduction from baseline to 6 months (D). Indeed, all statistical tests were 2-sided with a significance level set at *p* ≤ 0.05. Statistical analysis was performed using the R environment for statistical computing and graphics version 4.2 (R Foundation for Statistical Computing, Vienna, Austria; https://www.R-project.org/ (accessed on 21 May 2024)).

## Figures and Tables

**Table 1:** Post-operative MP-TLT-related complications are expressed as absolute frequencies.

Complications	Fs (n = 19)	Ss (n = 23)
Transient IOP spike *	2	1
Transient VA decrease ^§^	1	2
Conjunctival hyperemia	5	7
Conjunctival chemosis	2	4
Sub-conjunctival hemorrhage	1	0
Corneal abrasion	1	0
Anterior chamber inflammation	4	5
Eyelid hematoma	0	2
Macular edema	1	0

Fs: failures; Ss: successes. * IOP increase between 5 and 10 mmHg vs. baseline during the first postoperative week. ^§^ VA decrease ≥ 2 lines.

**Table 2:** Baseline and 6-month IOP and AS-OCT parameters in failed and successful MP-TLTs. *p*-value derived from the Wilcoxon rank sum test.

Variables	Fs			Ss		
	Baseline	6 Months	*p*-Value	Baseline	6 Months	*p*-Value
IOP	25.0 [22.0;30.5]	20.0 [18.0;24.5]	0.013	29.0 [26.0;34.5]	19.0 [14.0;21.5]	<0.001
I-MISHA	0.05 [0.04;0.07]	0.05 [0.04;0.06]	0.895	0.05 [0.03;0.08]	0.05 [0.04;0.06]	0.334
S-MISHA	0.03 [0.02;0.05]	0.06 [0.04;0.08]	0.037	0.03 [0.02;0.04]	0.06 [0.05;0.08]	<0.001
T-MISHA	0.05 [0.03;0.06]	0.05 [0.04;0.07]	0.358	0.04 [0.03;0.06]	0.06 [0.05;0.06]	0.102
I-SR	99.3 [89.8;106]	101 [89.8;103]	0.609	102 [95.2;113]	93.5 [86.5;98.4]	0.002
S-SR	97.7 [92.3;105]	96.4 [92.9;102]	0.511	106 [99.3;114] *	98.3 [90.4;107]	0.052
T-SR	97.6 [92.5;104]	98.0 [92.5;101]	0.422	104 [99.8;109] *	94.1 [90.9;101]	0.001

Fs: failures; Ss: successes. IOP (mmHg); MISHA (mm^2^); SR (arbitrary grading scale). *: differences between groups at baseline (S-SR: *p* = 0.031; T-SR: *p* = 0.033).

**Table 3:** Median differences in IOP and AS-OCT parameters between baseline and six months in failed versus successful MP-TLTs. *p*-values derived from the Mann–Whitney U test.

Variables	Fs (n = 19)	Ss (n = 23)	*p*-Value
DIOP	−5.00 [−7.00;−4.00]	−12.00 [−13.00;−10.00]	<0.001
DI-MISHA	0.00 [−0.02;0.01]	−0.01 [−0.02;0.01]	0.299
DS-MISHA	0.02 [−0.01;0.03]	0.03 [0.02;0.05]	0.061
DT-MISHA	0.00 [−0.01;0.02]	0.01 [0.00;0.02]	0.719
DI-SR	−0.28 [−14.53;9.37]	−10.43 [−18.06;0.15]	0.096
DS-SR	−0.83 [−9.41;6.37]	−2.76 [−9.14;−0.05]	0.598
DT-SR	−3.08 [−10.17;8.13]	−10.75 [−12.89;0.40]	0.188

Fs: failures; Ss: successes; D: difference (final value-baseline value). IOP: (mmHg); MISHA: (mm^2^); SR: (arbitrary grading scale).

## Data Availability

The data presented in this study are available on request from the corresponding author. The data are not publicly available due to privacy or ethical restrictions.
